# Distinct Glucose Profiles with Daily and Weekly Growth Hormone Therapy Revealed by Continuous Glucose Monitoring

**DOI:** 10.1210/jendso/bvaf193

**Published:** 2025-11-29

**Authors:** Tomomi Taguchi, Kaori Kimura, Satoshi Tsuji, Shiori Ito, Sara Takamura, Hideaki Shimotatara, Naoya Shimizu, Haremaru Kubo, Ayako Hoshiyama, Akinori Hayashi, Koji Takano, Takeshi Miyatsuka

**Affiliations:** Department of Diabetes, Endocrinology and Metabolism, Kitasato University School of Medicine, Sagamihara, Kanagawa 252-0374, Japan; Department of Diabetes, Endocrinology and Metabolism, Kitasato University School of Medicine, Sagamihara, Kanagawa 252-0374, Japan; Department of Diabetes, Endocrinology and Metabolism, Kitasato University School of Medicine, Sagamihara, Kanagawa 252-0374, Japan; Department of Diabetes, Endocrinology and Metabolism, Kitasato University School of Medicine, Sagamihara, Kanagawa 252-0374, Japan; Department of Diabetes, Endocrinology and Metabolism, Kitasato University School of Medicine, Sagamihara, Kanagawa 252-0374, Japan; Department of Diabetes, Endocrinology and Metabolism, Kitasato University School of Medicine, Sagamihara, Kanagawa 252-0374, Japan; Department of Diabetes, Endocrinology and Metabolism, Kitasato University School of Medicine, Sagamihara, Kanagawa 252-0374, Japan; Department of Diabetes, Endocrinology and Metabolism, Kitasato University School of Medicine, Sagamihara, Kanagawa 252-0374, Japan; Department of Diabetes, Endocrinology and Metabolism, Kitasato University School of Medicine, Sagamihara, Kanagawa 252-0374, Japan; Department of Diabetes, Endocrinology and Metabolism, Kitasato University School of Medicine, Sagamihara, Kanagawa 252-0374, Japan; Department of Fundamental Nursing, Kitasato University School of Nursing, Sagamihara, Kanagawa 252-0374, Japan; Department of Diabetes, Endocrinology and Metabolism, Kitasato University School of Medicine, Sagamihara, Kanagawa 252-0374, Japan; Department of Diabetes, Endocrinology and Metabolism, Kitasato University School of Medicine, Sagamihara, Kanagawa 252-0374, Japan

**Keywords:** growth hormone, adult growth hormone deficiency, somapacitan, growth hormone replacement therapy, continuous glucose monitoring

## Abstract

**Context:**

Somapacitan, a long-acting GH derivative, has been used to treat adult GH deficiency. Although the distinct pharmacokinetics of somapacitan compared with daily GH administration are presumed to affect daily and weekly glucose profiles, detailed information on glucose fluctuations remains limited.

**Objective:**

Glycemic variability in individuals during somapacitan treatment was assessed.

**Methods:**

A prospective, single-arm, single-center study was conducted in 20 adult GH deficiency participants without diabetes. Participants receiving somatropin were switched to somapacitan, with doses titrated over 3 months to maintain IGF-1 levels within ± 2 SD. Continuous glucose monitoring was performed for 14 days before and after the switch. The primary endpoint was the change in glycemic variability indices.

**Results:**

With IGF-1 levels matched between regimens, no significant differences were observed in glycated hemoglobin A1c, fasting blood glucose, and insulin resistance index before and after switching to somapacitan. The continuous glucose monitoring data demonstrated that the mean sensor glucose level (SGL) and continuous overall net glycemic action over 24 hours were significantly increased during somapacitan treatment. Notably, whereas SGLs showed no significant interday fluctuations during somatropin treatment, biphasic glucose fluctuations were observed during somapacitan treatment, with mean SGLs peaking on the second day and reaching their lowest on the fourth day. After switching to somapacitan, the time below range tended to decrease across all days.

**Conclusion:**

The switch from somatropin to somapacitan resulted in increased interday glucose variability, characterized by biphasic glucose fluctuations, suggesting a unique physiological effect of somapacitan on glucose profiles.

Adult GH deficiency (AGHD) is a rare endocrine disease caused by various hypothalamic-pituitary lesions, such as pituitary neuroendocrine tumors and postoperative complications [1]. Individuals with AGHD have been shown to demonstrate alterations in body composition, a dysregulated lipid profile, and decreased bone mass. GH replacement therapy (GHRT) has been demonstrated to reduce fat mass and total cholesterol levels, while increasing lean body mass and bone mineral density [2, 3]. In addition, AGHD is frequently associated with metabolic dysfunction-associated steatotic liver disease, which can be ameliorated by GHRT [4, 5]. AGHD also has a variable negative impact on quality of life, and GHRT can lead to an increase quality of life scores [6, 7]. Moreover, although AGHD is linked to a higher prevalence of cardiovascular diseases and increased mortality risk, GHRT can reduce the mortality risk [8].

Somapacitan is a long-acting GH derivative that can be administered once weekly for the treatment of AGHD. In 3 randomized controlled phase 3 trials (REAL 1, REAL 2, and REAL JP), no new cases of diabetes mellitus were reported among the participants treated with somapacitan [9-11]. Furthermore, although the weekly administration of GH derivatives is considered to differ substantially from the physiological pattern of endogenous GH secretion, post hoc analyses of these 3 trials demonstrated that somapacitan did not adversely affect glucose metabolism for up to 86 weeks in treatment-naïve or individuals previously treated for AGHD, compared with treatment with the daily GH formulation somatropin [12]. Although these clinical trials assessed glucose tolerance through fasting blood glucose levels and glycated hemoglobin A1c (HbA1c) levels, the detailed fluctuations in glucose profiles before and after switching from daily GH injections to somapacitan remain unclear.

Therefore, as the distinct pharmacokinetics of daily and weekly GH injections are thought to affect glucose profiles differently, this study aimed to clarify the detailed glucose profiles of participants both before and after switching from daily GH injections to somapacitan, using continuous glucose monitoring (CGM).

## Materials and Methods

### Study Design, Setting, and Participants

A prospective, single-arm interventional study was conducted at a single center. Twenty participants with AGHD, aged 20 to 60 years, who were seen at Kitasato University Hospital from August 26, 2021, to March 31, 2023, were included in this study. The diagnosis of AGHD was confirmed using the insulin tolerance test or the GH-releasing peptide-2 test [13]. The participants were scheduled to switch from daily GH injection therapy to somapacitan treatment. The exclusion criteria included a known history of diabetes mellitus, a known history of malignant tumors, pregnancy or likelihood of pregnancy, recent or scheduled surgery, and severe trauma. All the participants provided written informed consent. The primary endpoint was the difference in glycemic variability indices observed between the daily GH formulations and somapacitan treatment periods. A sample size of 15 participants was required to achieve 80% power with a Type I error of 0.05 and an effect size of 0.8. Considering potential participant dropout during the study, the final sample size was set at 20 participants. All methods were performed in accordance with the relevant guidelines and regulations of Kitasato University Hospital, as well as the Ethical Guidelines for Medical and Health Research Involving Human Subjects in Japan. The study protocol was approved by the Kitasato University Medical Ethics Organization (study approval number: KMEO C21-062; trial registration number: jRCT1031210321).

### Study Procedures

Before the study, the participants’ IGF-1 SD scores had been maintained within ± 2 SD by adjusting the dosage of their daily GH formulation. After confirming stable IGF-1 levels, the first CGM device (FreeStyle Libre Pro sensor; Abbott Diabetes Care, Alameda, CA, USA) was attached to continuously measure sensor glucose levels (SGLs) for 14 consecutive days. CGM recordings were considered valid if data were available for at least 70% of the 14-day observation period. After the first CGM device was removed, GHRT was switched from daily GH injections to somapacitan. Somapacitan was initiated at a dose of 1.5 mg/week in accordance with the prescribing information. During the 3-month titration period, IGF-1 SD scores were monitored on the fourth day after each somapacitan injection to adjust the dose and maintain the scores within the target range of ±2 SD. After the titration period, a second CGM device was attached, and sensor glucose levels were monitored for 14 days. Physical examinations and blood tests were performed on each participant before the attachment of the CGM devices.

### Data Collection and Measurement

Blood samples were collected after an overnight fast of at least 10 hours to measure levels of plasma glucose, immunoreactive insulin (IRI), C-peptide, HbA1c, glycated albumin, and IGF-1, as well as to perform lipid and liver function panels. During somapacitan treatment, blood samples were collected on the fourth day after each somapacitan injection. Homeostatic Model Assessment for Insulin Resistance (HOMA-IR), Homeostatic Model Assessment of β-cell Function, and C-peptide index were calculated using the following formulas: HOMA-IR = fasting blood glucose (mg/dL) × fasting IRI (µU/mL)/405, Homeostatic Model Assessment of β-cell Function = fasting IRI (μU/mL) × 360 ÷ (fasting blood glucose (mg/dL) 1-63), and C-peptide index = 100 × fasting C-peptide (ng/mL)/fasting blood glucose (mg/dL), respectively. Body composition parameters, including body weight, muscle mass, lean body mass, and fat mass were measured using an 8-electrode multifrequency bioelectrical impedance device (body composition analyzer MC-180; Tanita, Tokyo, Japan). Body mass index was calculated as body weight in kilograms divided by the square of height in meters.

SGL data were analyzed using EasyGV software [14], and the following metrics were calculated: mean, SD, coefficient of variation, continuous overall net glycemic action over 24 hours, high blood glucose index, low blood glucose index, percentage of time with SGLs between 70 and 180 mg/dL (time in range [TIR]), between 70 and 140 mg/dL (time in tight range [TITR]), <70 mg/dL (time below range; TBR^70^), <54 mg/dL (TBR^54^), >180 mg/dL (time above range [TAR]), and >140 mg/dL (time above tight range; TATR) [15]. Hypoglycemia was defined as SGL <70 mg/dL (3.9 mmol/L), and severe hypoglycemia as SGL <54 mg/dL [16]; hypoglycemia was additionally classified using the Ademolus Classification of Hypoglycemia (ACH) [17].

### Statistical Analysis

Statistical analyses were conducted using JMP Pro 17 software (SAS Institute, Inc., Cary, NC, USA) and GraphPad Prism 10.0.5 software (GraphPad Software Inc., San Diego, CA, USA). Data are presented as the mean ± SD, unless otherwise specified. The Friedman test, followed by the Dunn's multiple comparisons test or the Wilcoxon signed-rank test was used for statistical comparisons. The Fisher's exact test was used for categorical data analysis. In addition to nonparametric tests, linear mixed-effects models with participant-level random intercepts and fixed effects for treatment, adjusted for age, sex, and GH replacement dose were prespecified. A 2-tailed *P* value < .05 was considered to indicate a statistically significant difference between 2 groups.

## Results

### Characteristics of the Participants

Twenty participants with AGHD who were receiving daily GH injections and were scheduled to switch to the once-weekly GH derivative somapacitan were recruited for this study. Three of the 20 participants were excluded from the study because of either malfunction of their CGM device during the trial, which resulted in data loss, or the development of a severe illness not associated with the study. Therefore, a total of 17 participants completed the study and their data were analyzed. The clinical characteristics of the 17 participants are shown in Table 1. Among the participants with AGHD, the underlying causes included germinoma (n = 5), nonfunctioning pituitary neuroendocrine tumor (n = 3), craniopharyngioma (n = 2), pituitary stalk interruption syndrome (n = 1), Rathke's cleft cyst (n = 1), Sheehan's syndrome (n = 1), meningioma (n = 1), choriocarcinoma (n = 1), and unknown etiology (n = 2). None of the participants had active tumor lesions or isolated GH deficiency. All participants received hydrocortisone at a dose of 15 mg/day, except for 1 participant who did not receive hydrocortisone replacement therapy.

**Table 1.** Clinical background of the participants

Patient no.	Age (years)	Sex	Diagnosis	Dose of daily GH (mg/day)	Dose of weekly GH (mg/week)	Hydrocortisone (mg/day)	Hormone deficiency other than GHD
1	53	Male	PSIS	0.3	2.1	15	CAI, HH, CH
2	54	Male	Craniopharingioma	0.15	1	15	CAI, HH, CH, AVD
3	46	Male	Germinoma	0.2	1.5	15	CAI, HH, CH, AVD
4	46	Female	Craniopharingioma	0.15	1.5	15	CAI, CH, AVD
5	55	Male	Rathke's cleft cyst	0.2	1.5	15	CAI, HH, CH
6	43	Male	Germinoma	0.3	1.9	15	CAI, HH, CH, AVD
7	37	Male	Unknown	0.1	0.9	0	HH
8	55	Female	Choriocarcinoma	0.4	0.4	15	CAI, HH, CH
9	48	Male	NFPitNET	0.2	1.3	15	CAI, HH, CH
10	58	Female	Sheehan syndrome	0.2	1.5	15	CAI, CH
11	54	Female	NFPitNET	0.5	3	15	CAI, CH, AVD
12	25	Male	Germinoma	0.2	1.4	15	CAI, HH, CH, AVD
13	50	Female	Unknown	0.55	3.5	15	CAI, HH, CH
14	46	Male	NFPitNET	0.1	0.7	15	CAI, CH
15	34	Male	Germinoma	0.3	2	15	CAI, HH, CH, AVD
16	36	Male	Germinoma	0.225	1.5	15	CAI, HH, CH, AVD
17	51	Female	Meningioma	0.15	1.5	15	CAI, HH, CH, AVD

Abbreviations: AVD, arginine vasopressin deficiency; CAI, central adrenal insufficiency; CH, central hypothyroidism; HH, hypogonadotropic hypogonadism; NFPitNET, nonfunctioning pituitary neuroendocrine tumor; PSIS, pituitary stalk interruption syndrome.

### Effects of Switching from Once-daily to Once-weekly GH Derivatives on Participants’ Glucose Profiles and Other Clinical Parameters

There were no significant changes in body composition, fasting blood glucose, HbA1c, glycated albumin, and IGF-1 levels, IGF-1 SD score, or indices of insulin secretion and resistance after switching to somapacitan (Table 2). We also stratified the analysis by gender, which showed similar trends to those observed in the overall cohort, except for a slightly yet significantly higher body weight and body mass index in the female group during weekly GH treatment (Tables S1 and S2) [18]. A comparison of CGM parameters between treatment with daily and weekly GH injections demonstrated that mean SGLs were significantly higher during somapacitan treatment than during treatment with daily GH (Table 3; 105.0 ± 10.7 mg/dL vs 98.7 ± 10.1 mg/dL, *P* = .0046). Similarly, TATR (>140 mg/dL), TAR (>180 mg/dL), high blood glucose index, and maximum SGLs were significantly higher during somapacitan treatment. Notably, continuous overall net glycemic action over 24 hours was significantly increased by somapacitan treatment compared with during daily GH treatment (91.2 ± 10.0 mg/dL vs 86.0 ± 9.3 mg/dL, *P* = .020). No significant changes in both coefficient of variation and SD, which indicate diurnal fluctuations, were observed following the treatment switch. Glucose management indicator, which corresponds to the estimated HbA1c from CGM, was higher upon somapacitan treatment than upon daily GH treatment (5.28% ± 0.38% vs 5.06% ± 0.36%, *P* = .0038). Given the differences in participants’ backgrounds, we reanalyzed the SGL data using a mixed model adjusted for age, sex, and GH replacement dose. The results were consistent with those obtained in the unadjusted analysis (Table S3) [18]. Although no significant differences were observed in minimum SGLs between daily and weekly GH treatment, some participants experienced SGLs <54 mg/dL, which indicates moderate hypoglycemia in ACH grade 2 participants (Table S4) [18]. However, affected patients with low-glucose readings on CGM were all asymptomatic, and hypoglycemia was not confirmed by fasting venous sampling performed at a different time from the CGM measurements.

**Table 2.** Physical and laboratory data of the participants receiving daily or weekly GH injections

n = 17	Daily GH	Weekly GH	*P*
Weight (kg)	76.4 ± 18.7	76.7 ± 19.2	.35
BMI (kg/m^2^)	26.8 ± 5.3	26.9 ± 5.5	.26
Body fat (%)	30.3 ± 10.3	31.2 ± 9.9	.50
Fat mass (kg)	23.6 ± 11.6	24.8 ± 12.8	.72
Muscle mass (kg)	49.9 ± 13.5	49.0 ± 10.6	.72
Lean body mass (kg)	52.8 ± 14.1	51.8 ± 11.0	.70
Serum IGF-1 (ng/dL)	146 ± 43	154 ± 41	.37
Serum IGF-1 SD score	−0.34 ± 1.22	−0.06 ± 1.06	.32
AST (U/L)	24.2 ± 7.4	26.4 ± 12.4	.44
ALT (U/L)	24.7 ± 12.3	28.4 ± 20.4	.49
γ-GTP (U/L)	32.1 ± 26.0	33.2 ± 23.4	.67
ALP (U/L)	64.5 ± 23.0	71.8 ± 29.8	.081
LDH (U/L)	167 ± 41	176 ± 42	.**016**
Triglycerides (mg/dL)	130 ± 71	153 ± 96	.29
HDL cholesterol (mg/dL)	52.2 ± 16.4	57.1 ± 21.1	.060
LDL cholesterol (mg/dL)	104 ± 19	110 ± 22	.25
Fasting blood glucose (mg/dL)	97.2 ± 9.9	97.2 ± 10.3	.88
HbA1c (%)	5.70 ± 0.39	5.69 ± 0.38	.66
GA (%)	13.1 ± 1.9	13.2 ± 2.0	.85
IRI (U/L)	8.50 ± 4.62	10.24 ± 6.95	.22
HOMA-IR	2.08 ± 1.23	2.58 ± 1.92	.17
HOMA-β (%)	93.0 ± 48.6	102.6 ± 54.9	.78
CPR (ng/mL)	1.90 ± 0.85	2.00 ± 1.05	.93
CPI	1.94 ± 0.80	2.00 ± 0.91	.55

Data are presented as the mean ± SD. Statistical testing was performed using the Wilcoxon signed rank test between daily GH injections and weekly GH injections. Bold values indicate statistically significant differences.

Abbreviations: ALP, alkaline phosphatase; ALT, alanine aminotransferase; AST, aspartate aminotransferase; BMI, body mass index; CPI, C-peptide index; CPR, C-peptide; GMI, glucose management indicator; γ-GTP, gamma-glutamyl transpeptidase; HbA1c, hemoglobin A1c; HOMA-β, Homeostatic Model Assessment of β-cell Function; HOMA-IR, Homeostatic Model Assessment for Insulin Resistance; IRI, immunoreactive insulin; LDH, lactate dehydrogenase.

**Table 3.** Overall sensor glucose levels (SGL) of the participants receiving daily and weekly GH injections

n = 17	Daily GH	Weekly GH	*P*
Mean SGL (mg/dL)	98.7 ± 10.1	105.0 ± 10.7	.**0046**
SD (mg/dL)	21.7 ± 5.6	22.9 ± 6.0	.26
CV (%)	22.1 ± 5.8	22.1 ± 6.6	.92
CONGA_24_ (mg/dL)	86.0 ± 9.3	91.2 ± 10.0	.**020**
TIR (%)	90.9 ± 15.1	93.7 ± 11.3	.378
TITR (%)	85.9 ± 14.3	85.8 ± 12.1	.75
TBR^70^ (%)	8.5 ± 15.2	5.1 ± 11.2	.14
TATR (%)	5.6 ± 4.5	9.0 ± 7.1	.**0038**
TBR^54^ (%)	2.6 ± 5.1	1.4 ± 4.4	.11
TAR (%)	0.66 ± 1.56	1.17 ± 1.71	.**0024**
HBGI	0.99 ± 0.70	1.44 ± 0.98	.**0079**
LBGI	2.87 ± 2.98	2.6 ± 2.5	.89
Maximum SGL (mg/dL)	187 ± 30	201 ± 30	.**0066**
Minimum SGL (mg/dL)	50 ± 10	54 ± 11	.14
GMI (%)	5.06 ± 0.36	5.28 ± 0.38	.**0038**
HbA1c—GMI (%)	0.65 ± 0.27	0.41 ± 0.23	.**0020**

Data are presented as the mean ± SD. Statistical testing was performed using the Wilcoxon signed rank test between daily GH injections and weekly GH injections. Bold values indicate statistically significant differences.

Abbreviations: CONGA_24_, continuous overall net glycemic action over 24 hours; CV, coefficient of variation; GMI, glucose management indicator; HbA1c, hemoglobin A_1_c; HBGI, high blood glucose index; LBGI, low blood glucose index; SGL, sensor glucose level; TATR, time above tight range; TBR, time below range; TIR, time in range; TITR, time in tight range.

### Comparison of Glucose Variability Every 6 Hours and Interday between Once-daily and Once-weekly GH Treatments

CGM data analysis at 6-hour intervals demonstrated that the mean SGLs in participants treated with somapacitan were significantly higher than those receiving daily GH treatment at all measured time points (Table 4). Moreover, TATR (>140 mg/dL) was significantly higher in participants treated with somapacitan than with daily GH treatment, particularly during the time intervals of 00:00 to 05:59, 12:00 to 17:59, and 18:00 to 23:59. The minimum SGLs dropped below 54 mg/dL between 00:00 and 05:59 in some patients receiving either daily or weekly GH treatment.

**Table 4.** Sensor glucose levels in six-hour intervals of the participants receiving daily GH or weekly GH injections

n = 17	0:00-5:59			6:00-11:59		
	Daily GH	Weekly GH	*P*	Daily GH	Weekly GH	*P*
Mean SGL (mg/dL)	88.5 ± 12.6	94.6 ± 13.4	.**017**	93.3 ± 12.5	98.9 ± 11.5	.**040**
TIR (%)	85.5 ± 26.5	90.9 ± 19.1	.12	88.0 ± 21.8	93.3 ± 16.1	.**043**
TITR (%)	84.7 ± 26.2	88.7 ± 18.7	.43	85.4 ± 20.3	89.4 ± 16.3	.43
TBR^70^ (%)	14.5 ± 26.5	8.9 ± 19.2	.12	11.7 ± 21.9	6.3 ± 15.8	.**048**
TATR (%)	0.8 ± 1.5	2.3 ± 3.2	.**0039**	3.1 ± 3.8	4.3 ± 5.1	.24
TBR^54^ (%)	4.5 ± 8.2	3.1 ± 9.1	.21	3.5 ± 7.6	1.8 ± 6.3	.16
TAR (%)	0.0 ± 0.0	0.2 ± 0.6	1.00	0.2 ± 0.7	0.4 ± 0.6	.30

	12:00-17:59			18:00-23:59		
	Daily GH	Weekly GH	*P*	Daily GH	Weekly GH	*P*
Mean SGL (mg/dL)	106 ± 13	112 ± 15	.**011**	107 ± 11	114 ± 11	.**00020**
TIR (%)	93.8 ± 8.5	94.4 ± 7.8	.86	94.1 ± 10.0	96.1 ± 5.6	.77
TITR (%)	86.4 ± 10.3	82.1 ± 14.3	.16	85.7 ± 11.0	83.0 ± 11.4	.19
TBR^70^ (%)	4.6 ± 8.0	3.0 ± 6.1	.47	5.0 ± 10.1	2.3 ± 5.0	.14
TATR (%)	8.9 ± 9.9	14.8 ± 14.6	.**016**	9.2 ± 6.8	14.7 ± 10.8	.**0052**
TBR^54^ (%)	1.5 ± 3.7	0.4 ± 1.2	.22	1.7 ± 3.3	0.4 ± 1.3	.063
TAR (%)	1.6 ± 4.0	2.5 ± 4.9	.**039**	0.8 ± 2.0	1.6 ± 1.9	.061

Data are presented as the mean ± SD. Statistical testing was performed using the Wilcoxon signed rank test between daily GH injections and weekly GH injections. Bold values indicates statistically significant differences.

Abbreviations: CONGA_24_, continuous overall net glycemic action over 24 hours; SGL, sensor glucose level; TATR, time above tight range; TBR, time below range; TIR, time in range; TITR, time in tight range.

**Figure 1.**
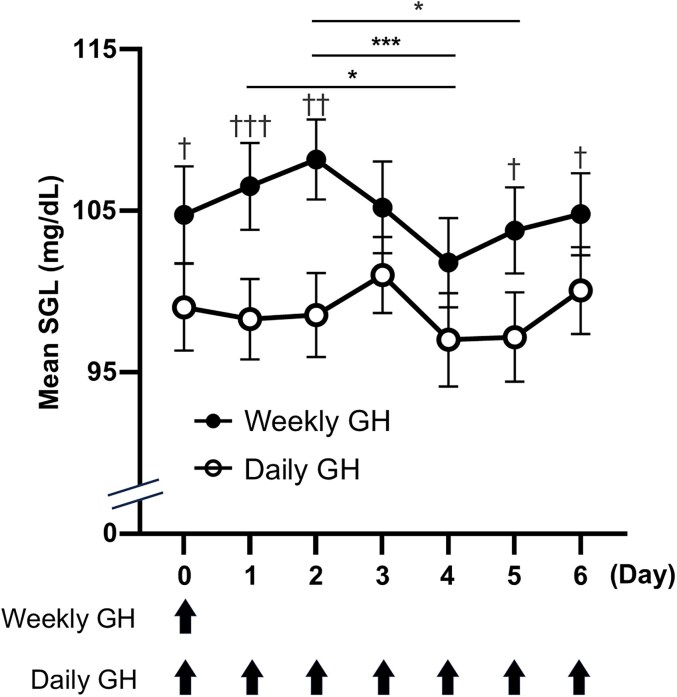
Mean SGLs of participants receiving either daily or weekly GH injections over a 7-day period. Graph showing the mean SGLs over a 7-day period of participants receiving either daily GH (open circles) or weekly GH (filled circles) injections. Data are presented as the mean ± standard error (SE). Statistical testing was performed using the Friedman test, followed by the post hoc Dunn's test (*P < .05, ***P < .001) or Wilcoxon signed-rank test (†*P* < .05, ††*P* < .01, †††*P* < .001). Abbreviation: SGL, sensor glucose level.

**Figure 2.**
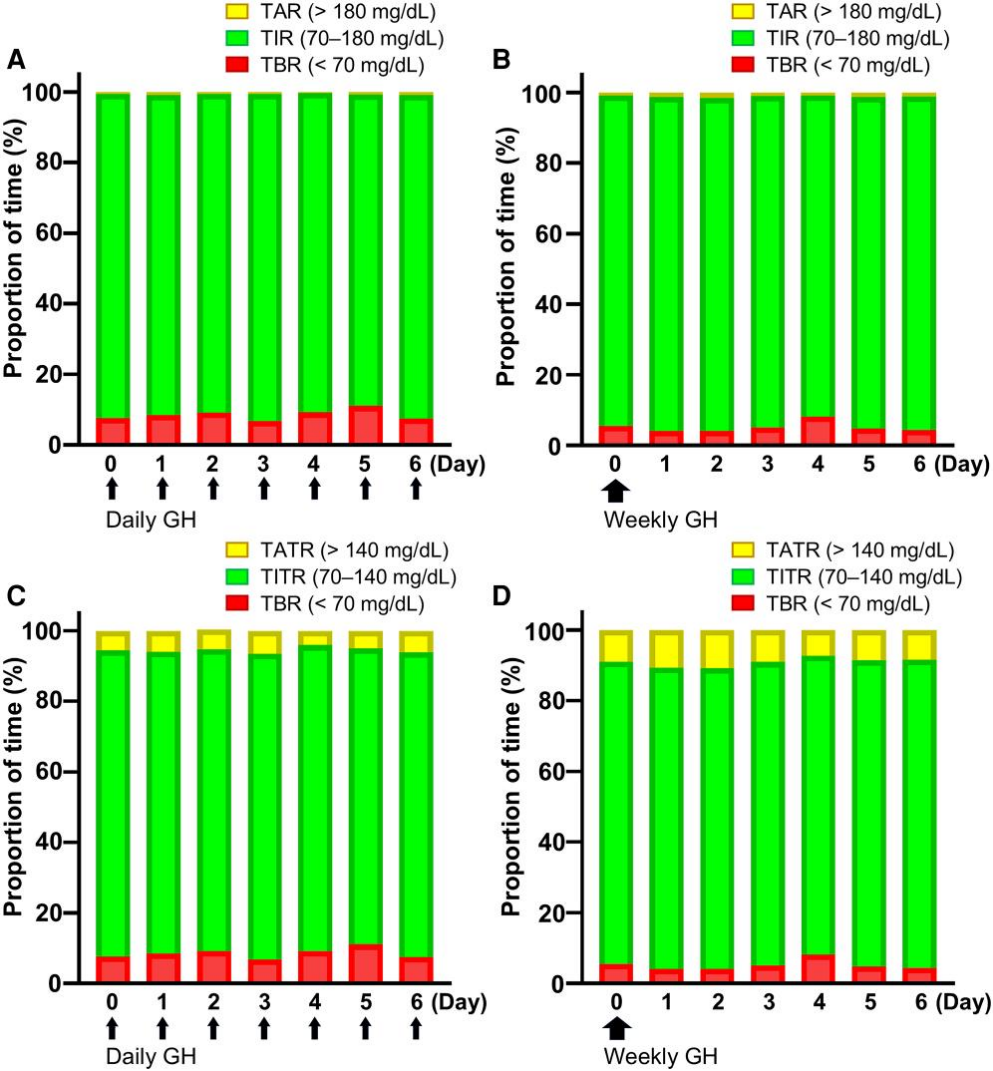
CGM metrics of patients receiving either daily or weekly GH injections over a 7-day period. (A, B) Proportion of time in TAR (>180 mg/dL), TIR (70-180 mg/dL), and TBR (<70 mg/dL) in participants undergoing either daily (A) or weekly GH injections (B) over a 7-day period. (C, D) Proportion of time in TATR (>140 mg/dL), TITR (70-140 mg/dL), and TBR (<70 mg/dL), in participants undergoing either (C) daily or (D) weekly GH injections over a 7-day period. Abbreviations: SGL, sensor glucose level (values in mg/dL); TAR, time above range; TATR, time above tight range; TBR, time below range; TIR, time in range, TITR, time in tight range.

To further clarify the temporal dynamics of the glycemic fluctuations, we analyzed CGM data on a daily basis (Table 5, Figs. 1 and 2). Mean SGLs in participants treated with somapacitan were significantly higher than those receiving daily GH treatment on days 0, 1, 2, 5, and 6, whereas no significant difference was observed on days 3 and 4. This pattern suggests a potential biphasic response in glucose profiles to the weekly GH derivative. This biphasic glycemic pattern was characterized by a peak in mean SGLs on day 2 (108 ± 10.0 mg/dL), followed by a nadir on day 4 (102 ± 11.4 mg/dL). Notably, after reaching this nadir, the mean SGLs began to increase again from day 4 onwards, then returning to levels nearly identical to those on day 0 by day 6. Thus, somapacitan treatment affects interday glucose profiles in a manner distinctly different from those observed during daily GH treatments.

**Table 5.** Interday sensor glucose levels of the participants receiving daily and weekly GH injections

n = 17	Day 0			Day 1		
	Daily GH	Weekly GH	*P*	Daily GH	Weekly GH	*P*
Mean SGL (mg/dL)	99.0 ± 11.0	105 ± 12	.**020**	98.3 ± 10.3	107 ± 11	.**00030**
TIR (%)	91.8 ± 14.1	93.7 ± 12.2	.29	92.4 ± 14.4	94.5 ± 12.9	.50
TITR (%)	86.9 ± 12.8	85.5 ± 12.0	.54	82.8 ± 22.1	85.3 ± 14.4	.38
TBR^70^ (%)	7.6 ± 14.2	5.5 ± 12.4	.11	8.5 ± 15.9	4.1 ± 12.5	.12
TATR (%)	5.5 ± 5.1	8.9 ± 7.6	.**0067**	5.9 ± 6.5	10.6 ± 8.2	.**0017**
TBR^54^ (%)	2.0 ± 5.4	1.1 ± 3.4	.13	5.0 ± 15.6	1.1 ± 3.9	.31
TAR (%)	0.6 ± 1.5	0.8 ± 1.8	.50	3.0 ± 8.9	1.4 ± 2.2	.31

	Day 2			Day 3		
	Daily GH	Weekly GH	*P*	Daily GH	Weekly GH	*P*
Mean SGL (mg/dL)	98.6 ± 10.7	108 ± 10	.**0021**	101 ± 10	105 ± 12	.16
TIR (%)	90.3 ± 17.5	94.3 ± 12.4	.62	92.7 ± 13.8	93.9 ± 12.4	.91
TITR (%)	85.6 ± 16.6	85.2 ± 14.9	.40	86.7 ± 13.1	85.8 ± 13.6	.70
TBR^70^ (%)	9.1 ± 17.6	4.0 ± 11.2	.15	6.8 ± 13.9	5.1 ± 12.6	.57
TATR (%)	5.7 ± 4.5	10.8 ± 8.8	.**043**	6.5 ± 5.3	9.1 ± 8.2	.17
TBR^54^ (%)	3.0 ± 6.7	1.7 ± 7.2	.63	2.0 ± 7.0	.7 ± 2.1	1.00
TAR (%)	0.6 ± 1.2	1.6 ± 2.7	.**035**	0.6 ± 1.4	1.0 ± 1.6	.27

	Day 4			Day 5		
	Daily GH	Weekly GH	*P*	Daily GH	Weekly GH	*P*
Mean SGL (mg/dL)	97.0 ± 11.9	102 ± 11	.057	97.2 ± 11.4	104 ± 11	.**015**
TIR (%)	90.4 ± 19.0	90.8 ± 14.1	.90	88.2 ± 18.3	93.9 ± 9.5	.19
TITR (%)	86.7 ± 18.0	84.5 ± 14.4	.38	83.9 ± 18.4	86.8 ± 10.9	.63
TBR^70^ (%)	9.3 ± 19.2	8.2 ± 14.0	.94	11.0 ± 18.2	4.8 ± 9.8	.**033**
TATR (%)	4.0 ± 4.2	7.3 ± 6.2	.**0058**	5.1 ± 4.6	8.5 ± 7.7	.**019**
TBR^54^ (%)	2.9 ± 5.9	3.0 ± 7.9	.64	4.6 ± 9.9	1.6 ± 5.0	.063
TAR (%)	0.3 ± 0.9	0.9 ± 1.7	.**031**	0.7 ± 2.0	1.3 ± 2.2	.13

	Day 6					
	Daily GH	Weekly GH	*P*			
Mean SGL (mg/dL)	100.0 ± 11	105 ± 10	.**020**			
TIR (%)	91.6 ± 15.8	94.4 ± 9.6	.94			
TITR (%)	86.4 ± 15.9	87.1 ± 10.5	.46			
TBR^70^ (%)	7.5 ± 15.2	4.4 ± 9.4	.38			
TATR (%)	6.1 ± 6.2	8.4 ± 6.7	.065			
TBR^54^ (%)	1.9 ± 5.8	0.8 ± 2.4	.31			
TAR (%)	0.9 ± 2.8	1.2 ± 1.7	.19			

Data are presented as the mean ± SD. Statistical testing was performed using the Wilcoxon signed rank test between daily GH injections and weekly injections for each day. Bold values indicate statistically significant differences.

Abbreviations: CONGA_24_, continuous overall net glycemic action over 24 hours; TATR, time above tight range; TBR, time below range; TIR, time in range; TITR, time in tight range.

## Discussion

In this study, a comparison of continuous SGL measurements between individuals with AGHD receiving daily and weekly GH treatments demonstrated unexpected glucose fluctuations associated with weekly somapacitan treatment. Previous studies have shown comparable glucose profiles between daily GH and somapacitan treatment [10, 12]. Whereas our study showed similar HbA1c and fasting blood glucose levels between daily and weekly GH treatments, our CGM data demonstrated a slight but significant increase in mean SGLs following somapacitan treatment across all days of the 2-week observation period. This suggests that glucose management based on fasting blood glucose and HbA1c, as well as the dose titration of GH derivatives guided by IGF-1 levels, does not provide an accurate assessment of the overall glucose profile, highlighting the clinical usefulness of CGM metrics in evaluating glucose dynamics.

During GHRT with somapacitan, the mean SGL peaked on day 2 and reached its lowest point on day 4 (Fig. 1). Previous studies have shown that blood levels of somapacitan peak 9 hours after its injection, followed by a gradual decrease in concentration [19]. Injected IGF-1 acts like insulin in reducing lipolysis [20, 21], and it lowers blood glucose in rodents and humans [22-24]. In children or young adults with extreme insulin resistance syndrome, IGF-1 administration has been reported to increase IGF-1 levels to an average of 349 ng/mL, accompanied by a reduction in blood glucose of more than 100 mg/dL [25]. In adult subjects with normal renal function, somapacitan reaches its peak plasma concentration within 12 hours of administration, and then gradually decreases, whereas IGF-1 levels peak at approximately 300 ng/mL about 72 hours after its administration [26]. These observations suggest that the mechanism of biphasic changes in SGLs observed in this study following somapacitan administration result from a temporal disparity between blood somapacitan levels and endogenous IGF-1 peaks. Specifically, the initial effect is attributed to the direct action of somapacitan as a GH derivative on insulin resistance, leading to peak SGLs on day 2. Subsequently, a delayed effect mediated by increased IGF-1, which is indirectly induced by somapacitan, results in a significant decrease in SGLs 4 days after somapacitan administration. From day 4 onward, the opposing effects of GH and IGF-1 appear to counterbalance each other, gradually returning blood glucose levels to baseline. Because this study included only participants with normal glucose tolerance, the increase in SGL, which peaked on day 2 during weekly GH treatment, was not sufficient to induce diabetes mellitus. However, in individuals with diabetes, there may be a risk of experiencing days of marked hyperglycemia during weekly GH treatment. Based on our findings, we believe that attention should be paid to glucose fluctuations in patients receiving weekly GH therapy.

Despite a decrease in SGL on day 4 of somapacitan treatment, SGL remained higher than that observed during daily GH treatment. At this point, IGF-1 levels were comparable between regimens, whereas HOMA-IR tended to be higher in participants undergoing somapacitan treatment. Although a direct comparison of blood concentrations of somapacitan and somatropin on day 4 was not feasible, SGL also remained higher in participants undergoing somapacitan treatment on day 6, when somapacitan levels were expected to be near the trough. This suggests that factors other than somapacitan's insulin-antagonistic action contribute to the increase in SGL.

Our CGM findings support the concept that pharmacokinetic differences between long-acting and daily GH formulations can yield discordant physiological effects across endpoints. For example, a study demonstrated that in patients with lifelong isolated GH deficiency, 6 months of treatment with a depot GH preparation improved body composition and lipid profile without affecting HbA1c level, yet was accompanied by a progressive increase in carotid intima-media thickness and atherosclerotic plaques [27]. In addition, evidence supporting the use of long-acting GH in cancer survivors remains insufficient [28]. Considering our observation of greater interday glycemic variability with once-weekly GH treatment than with once-daily GH treatment, CGM-based monitoring may be crucial, particularly in individuals with diabetes or those with a high risk of cardiovascular disease. Prospective studies specifically designed to evaluate long-term glycemic variability, vascular outcomes, and oncologic safety in users of long-acting GH are warranted.

Interestingly, some participants experienced TBR^54^, which indicates severe hypoglycemia. The frequency of TBR^54^ was highest during the 0:00 to 5:59 period in both participants receiving once-daily and once-weekly GH injections. Previous studies using CGM in patients with central adrenal insufficiency have reported that hypoglycemia can occur despite hydrocortisone replacement therapy [29]. Although none of the participants in the present study experienced hypoglycemic episodes accompanied by counterregulatory responses in the central nervous system throughout the study period, it is possible that they repeatedly experienced hypoglycemia without being aware of it. In contrast, CGM measures interstitial glucose rather than blood glucose, and method-associated discrepancies may occur, particularly at low glucose levels. Indeed, among the participants with low-glucose readings on CGM, hypoglycemia was not confirmed by fasting venous sampling performed at time points distinct from the CGM measurements. This finding underscores the uncertainty regarding the true frequency of unrecognized hypoglycemia in this cohort. After switching to somapacitan, both TBR^70^ and TBR^54^ tended to be lower overall, although between-participant heterogeneity and interday fluctuations were evident (Tables 4 and 5). Notably, a subset of participants demonstrated TBR^54^ under both treatments (Table S4) [18], indicating the existence of a subgroup with a persistently higher low-glucose burden regardless of regimen. Whether switching to somapacitan truly reduces TBR and mitigates hypoglycemia warrants cautious interpretation.

This study has several limitations. First, because we did not collect dietary records from the participants, we were unable to assess any changes in food intake associated with the transition from daily to weekly GH replacement therapy. It is possible that an increase in food intake during weekly GH administration contributed to the increase in SGLs. However, as significant weight gain was not observed in the participants during the study period, this possibility appears to be minimal. Moreover, even during the overnight period (00:00-05:59), which is less affected by food intake, mean SGL and TATR were higher in the weekly GH treatment period than in the daily GH period, consistent with the whole-day results. Taken together, the observed changes in glucose profiles were more likely attributable to switching from daily to weekly GH than to dietary influences. Second, given the small sample size and relatively short follow-up period (only 3 months), it is difficult to draw conclusions regarding the long-term effects of switching to somapacitan on glucose profiles. To address these limitations, future studies should analyze sensor glucose profiles using CGM over extended periods in larger populations of patients undergoing weekly GH injections. Third, this study lacked a control group and had a small sample size, limiting external validity and making it difficult to distinguish the treatment effect from temporal or carryover effects. Future randomized crossover or parallel-group trials with appropriate controls are required to confirm these findings. Last, as the CGM used in this study does not report SGL values <40 mg/dL, we were unable to assess individuals with ACH grades 3 and 4. Moreover, CGM is less accurate in the hypoglycemic range. To address these issues, the presence and severity of hypoglycemia should be confirmed by either nocturnal venous blood sampling or the self-monitoring of blood glucose.

In summary, we found that somapacitan induces biphasic fluctuations in interday SGL variability, suggesting novel physiological effects of GH and IGF-1 on glucose metabolism. Furthermore, we showed that switching from once-daily to once-weekly GH injections leads to an increase in SGLs and a reduction in the duration of hypoglycemia. Future studies with an extended observation period and a larger cohort of people with AGHD would provide further insights into the clinical significance of weekly GH injections on glucose stability.

## Data Availability

The datasets analyzed in this study are available from the corresponding author upon reasonable request.

## Clinical Trial Information

**Clinical Trial Registration number:** jRCT1031210321.

## Abbreviations

ACH: Ademolus Classification of Hypoglycemia
AGHD: adult GH deficiency
CGM: continuous glucose monitor
GHRT: GH replacement therapy
HbA1c: glycated hemoglobin A1c
HOMA-IR: Homeostatic Model Assessment for Insulin Resistance
IRI: immunoreactive insulin
SGL: sensor glucose level
TAR: time above range
TATR: time above tight range
TBR: time below range
TIR: time in range
TITR: time in tight range
